# Health literacy and fatigue, anxiety, depression, and somatic symptoms in patients with differentiated thyroid carcinoma from West China: A cross‐sectional study

**DOI:** 10.1002/hsr2.1018

**Published:** 2023-01-10

**Authors:** MengMeng Huang, YunJian Wu, XianXiu Wen, WenZhong Song

**Affiliations:** ^1^ Department of Nursing Sichuan Provincial Hospital Chengdu China; ^2^ Cancer Programme, Biomedicine Discovery Institute Monash University Clayton Victoria Australia; ^3^ Department of Biochemistry and Molecular Biology Monash University Clayton Victoria Australia; ^4^ Department of Nuclear Medicine Sichuan Provincial Hospital Chengdu China

**Keywords:** differentiated thyroid carcinoma, health literacy, health‐related quality of life

## Abstract

**Background and Aims:**

Differentiated thyroid carcinoma (DTC) patients are associated with excellent prognosis but impaired health‐related quality of life (HRQOL) by initial and subsequent therapy. Health literacy plays a pivotal role in public health and medical settings, but data on its relationship with DTC patients' HRQOL are insufficient and equivocal. This study was designed to explore the relationship between health literacy and HRQOL in patients with DTC from West China areas.

**Methods:**

A cross‐sectional study with a descriptive correlational design was conducted. 126 patients with DTC were recruited between 2020 and 2021. Levels of health literacy and HRQOL (including fatigue level, anxiety/depression status, and somatoform symptoms) were assessed by questionnaires. Pearson product–moment correlation and Stepwise multiple regression were used to examined the adjusted association of health literacy with HRQOL.

**Results:**

Health literacy and receiving DTC‐related education together explained 16.2% of the variance in fatigue level. Patients who had higher health literacy, received more DTC‐related education were currently employed and less fatigue. Health literacy, fatigue level and DTC‐related education together explained 31.0% of the variance in anxiety and depression of DTC patients. Patients who had higher health literacy, received more DTC‐related education and less fatigue level were less anxious and depressive. Age explained 8.3% of the variance in DTC patients' somatoform symptoms. Older patients complained more somatoform symptoms.

**Conclusion:**

Health literacy was positively associated with HRQOL in DTC patients regarding to fatigue level and anxiety/depression status across the entire sample. Interventions to improve HRQOL should take the patients' health literacy into account.

## 1 INTRODUCTION

In recent decades, the incidence of differentiated thyroid cancer (DTC) has dramatically increased worldwide, mostly due to the advantages of thyroid gland imaging techniques and other diagnostic methods.^1^, ^2^ With incidence increasing every year, now DTC is the fifth most common cancer diagnosed in women in the United States in 2017,^3^ and this trend can also be observed in China.^4^ Papillary thyroid carcinoma and follicular thyroid carcinoma are the most common DTC, and its standard treatment includes thyroid surgery (either total thyroidectomy or hemithyroidectomy), for some, followed by radioiodine (131I) thyroid remnant ablation therapy and require thyroid hormone replacement. Most DTC patients can be cured by proper treatment, with approximately 90%–95% 10‐year survival rate.^5^

Although the good prognosis and moderate invasiveness of the common therapies, the health‐related quality of life (HRQOL)^6^, ^7^ is still negatively affected in patients with DTC compared with general population.^8^, ^9^, ^10^ Of note, the overdiagnosis of low‐risk DTC has raised concerns recently, and the associated overtreatment also brought substantial impacts on the HRQOL of these patients.^1^, ^11^ For example, all the therapeutic options described above possess potential adverse effects and consequences, including vocal cord paralysis, hypoparathyroidism after thyroidectomy, and hyperthyroidism after supraphysiologic doses of thyroid hormone to suppress thyrotropin levels.^12^ A couple of comparative studies found multiple aspects of HRQOL can be impaired by these treatments, including vitality, role‐physical, mental health, role‐emotional and social functioning.^8^, ^9^, ^10^, ^13^

Health literacy is “the degree to which individuals have the capacity to obtain, process and understand basic health information and services needed to make appropriate health decisions,”^14^ defined by the US Department of Health and Human Services (HHS) and the National Academy of Medicine (NAM). Health literacy is one of the most important determinants of health and medical outcomes. Poor or inadequate health literacy results in difficulties in understanding health information, limited knowledge of diseases, increased risk for emergency care and hospitalizations, poor medication adherence, higher mortality rates, increased costs, and health disparities.^14^, ^15^, ^16^ Increasing studies have shown that health literacy could directly or indirectly influence the HRQOL of patients. For example, health literacy was shown to be significantly associated with self‐care skill and self‐efficacy in patients with diabetes,^17^ and limited health literacy was correlated with a poorer social quality of life in head and neck cancer patients.^18^ However, other reports revealed that adjusting health literacy did not change self‐efficacy and self‐management associations in diverse low‐income populations in urban areas,^19^ and no significant relationship between health literacy and HRQOL among patients with rheumatic diseases.^20^ Despite the inconsistent findings in the literature, health literacy is needed for all people, for instance, to find their way to the right place in a hospital, fill out medical and insurance claim forms, and communicate with physicians and nurses. Health literacy is also important to DTC patients, since limited health literacy may lead to difficulties in interpreting thyroid stimulating hormone (TSH), free thyroxine 4 (FT4), free thyroxine 3 (FT3), and thyroglobulin (Tg) reading. It will also lead to poor performance in adjusting medication, avoiding iodized food intake, and performing other self‐care activities. Currently, the relationship between health literacy and HRQOL is still poorly defined. Nickel et al^21^ identified that patients diagnosed with DTC reported wide‐ranging HRQOL issues, but the correlation with health literacy was still unclear. Therefore, here we perform this study to decipher the association between health literacy and HRQOL in patients with DTC.

## 2 METHODS

### 2.1 Participants and setting

Our study relied on patients diagnosed with DTC who were initially received total thyroidectomy or hemithyroidectomy, and admitted to nuclear medicine department for postoperative radioiodide ablation therapy with I‐131 in Sichuan Provincial Hospital between 2020 and 2021. A convenience sample of 126 DTC patients was recruited. Inclusion criteria were: a) age between 16 and 70, b) confirmed DTC diagnosis, and c) ability to speak and understand Mandarin. Patients were excluded with organ metastasis, impaired vision, psychiatric illness or dementia. Initially, 130 eligible DTC patients were recruited. Four patients dropped out due to time constraints (*n* = 3) and inability to complete the questionnaires used in this research (*n* = 1).

### 2.2 Ethical considerations

This study was approved by the Ethical Committee of Sichuan Provincial People's Hospital (IRB No. CTR2020321). Each participant received an oral explanation of the research, and we received inform consent from all individuals who agreed to participate in the study. All data will be held securely in accordance with Data Protection Regulations. Patient confidentiality will be maintained at all times. Findings will be widely disseminated in peer‐reviewed journals, at conferences, through user networks, and to policy makers and relevant clinical groups.

### 2.3 Measurements

#### 2.3.1 Socio‐demographic characteristics

The following socio‐demographic characteristics of patients were recorded: age, gender, ethnicities, educational level, marital status, employment status, living arrangement, and income level. The section of ‘highest attained educational level’ falls into five categories including: no formal schooling, elementary school (Grade 1–6), junior high school (Grade 7–9), high school (Grade 10–12), and college and above. The attendance of DTC‐related teaching classes held by Sichuan Provincial People's Hospital in the past 12 months was also recorded.

#### 2.3.2 Health literacy

The health literacy was measured by the scale of “Health Literacy for Chinese Citizens‐Basic Knowledge and Skills (HLCCBKS)”, which was developed by Chinese Ministry of Health in 2008.^22^, ^23^ This 66‐item questionnaire addresses three dimensions: 25 items of basic knowledge and belief, 34 items of health lifestyle and behavior, and 7 items of basic skills. Scores are summed to give a total range from 0 to 66; higher scores represent greater health literacy. In the current study, Cronbach's alpha coefficient was 0.82 for the overall scale, and for the three dimensions (basic knowledge and belief, health lifestyle and behavior, and basic skills), it was 0.81, 0.78, and 0.86, respectively.

#### 2.3.3 HRQOL questionnaires

Three health‐related questionnaires that were validated by previous studies were used to measure the HRQOL of DTC patients.

1. Multidimensional Fatigue Index‐20 (MFI‐20)
  Since posttreatment fatigue is one of the most common and important complaints in DTC patients,^24^ the MFI‐20 scale was employed in this research. The MFI‐20 comprises 20 statements to assess fatigue, which are measured on a 5‐point scale.^25^ The domain‐specific MFI‐20 measures fatigue in five dimensions: general fatigue, physical fatigue, reduction in activity, reduction in motivation, and mental fatigue. Scale scores from 4 to 20. The higher score indicates the more fatigue. The Cronbach's alphas were 0.86 in the current study.
2. Hospital Anxiety and Depression Scale (HADS)
  The HADS is a well‐established self‐report tool to understand the emotional distress and anxiety in the setting of medical practice,^26^, ^27^ and is frequently utilized in various cancers,^28^, ^29^, ^30^ and other diseases.^31^, ^32^ The HADS contains 14 items with seven to assess anxiety and seven to assess depression. Scores for the anxiety and depression subscale range from 0 to 21, and the score is interpreted as: 0–7, no depression or anxiety disorder; 8–10, mild depression or anxiety disorder; 11–14, moderate disorder; and 15–21, severe disorder. In the present study, Cronbach's alphas were 0.78.
3. Screening for Somatoform Symptoms‐7 (SOMS‐7)
  This questionnaire covers all somatic symptoms mentioned as occurring in somatization disorder, according to Diagnostic and Statistical Manual of Mental Disorders fourth edition (DSM‐Ⅳ) and International Statistical Classification of Diseases and Related Health Problem‐10 (ICD‐10).^33^ The scale (51 items for men and 53 items for women) assesses the severity of signs and symptoms according to the grading criteria, which is from 0 for the lowest intensity to 4 for the maximum intensity. The total score expresses the extent of physical complaints presented in the last seven days. The Cronbach's alphas were 0.86 in the current study.

### 2.4 Data collection

A trained project investigator (M.M.H.) approached potential participants during their initial admission to the nuclear medicine ward. Potential participants were screened for inclusion and exclusion criteria. If eligible, the investigator explained the study purposes and procedures, and the participant's rights to participate or to refuse participation without jeopardizing medical care. Individuals who were interested in participating in the study signed an informed consent that explained study procedures and assured confidentiality. After obtaining informed consent, participants were asked to complete the self‐report questionnaires.

### 2.5 Data analysis

The participants' demographics and study features were described using percentages, means, and standard deviations (SDs). The relationships among demographics (age, gender, marital status, education level, employment status, and living arrangement), health literacy and HRQOL were examined by Independent 2‐sided *t*‐tests, one‐way analysis of variance (ANOVA), and Pearson product–moment correlations. Stepwise multiple regression analysis was used to sequentially identify the key predictive factors by correlating demographics, disease characteristics (DTC education), health literacy and the HRQOL (assessed by MFI‐20, HADS, and SOMS‐7). All statistical analyses were performed with the SPSS Version 17.0 software statistical package (IBM). A *p*‐value < 0.05 was considered as statistically significance.

## 3 RESULTS

### 3.1 Participant characteristics

Totally, 126 participants were recruited, of which 103 (81.75%) were female, and 23 were male (18.25%). The mean age of participants was 43.32 years (SD = 11.31; range = 16–65). More participants were married (79.37%), employed (71.43%), and living with family (92.06%) in this patient cohort. The educational level ranged from ‘never attended school’ to “postcollege.” The majority (88.09%) of participants had a junior high school or above education level. Frequency (times per year) of patients receiving DTC education ranged from 0 to 7, with a mean 3.14 (SD = 1.51). Of note, 7 (5.56%) participants indicated that they never received or gained information about DTC or risk factors from either doctors or nurses. The main sociodemographic characteristics of the samples were summarized Table 1.

**Table 1:** Demographic characteristics of the sample and comparisons of health literacy, and HRQOL among different characteristics

		Health literacy		MFI‐20 score		HADS score		SOMS‐7 score	
Characteristic	*n* (%)	Mean (SD)	*t/F*	Mean (SD)	*t/F*	Mean (SD)	*t/F*	Mean (SD)	*t/F*
Gender			−1.14		0.46		−0.93		−1.03
Female	103 (81.75)	33.89 (6.08)		37.27 (4.51)		10.12 (4.18)		10.71 (4.12)	
Male	23 (18.25)	35.52 (6.59)		36.78 (5.04)		11.00 (3.95)		11.78 (6.03)	
Marital status			−0.71		1.98		0.92		1.20
Married	100 (79.37)	33.99 (6.52)		37.51 (4.84)		10.45 (4.17)		11.15 (3.89)	
Single	26 (20.63)	34.96 (4.72)		35.92 (3.27)		9.62 (4.00)		9.96 (6.40)	
Educational level			1.923		1.49		1.85		1.27
Never attend school	2 (1.59)	25.50 (10.61)		40.00 (4.24)		15.00 (8.49)		9.50 (2.12)	
Elementary	13 (10.32)	32.62 (8.34)		35.38 (4.59)		9.00 (4.16)		11.15 (4.06)	
Junior high school	49 (38.89)	33.71 (5.99)		37.84 (3.89)		10.22 (4.15)		11.90 (4.10)	
High school	46 (36.51)	34.76 (5.86)		37.43 (5.08)		9.15 (3.90)		10.33 (4.84)	
College and above	16 (12.69)	36.38 (4.32)		35.56 (4.86)		10.81 (4.05)		9.44 (5.10)	
Employment status			3.17**		0.32		−0.14		0.45
Employed	90 (71.43)	35.26 (6.00)		37.27 (4.85)		10.24 (4.08)		11.01 (4.26)	
Unemployed	36 (28.57)	31.53 (5.90)		36.97 (3.92)		10.36 (4.33)		10.61 (5.18)	
Living arrangement			0.95		0.85		0.30		−1.54
Living with someone	116 (92.06)	34.34 (5.96)		37.28 (4.65)		10.31 (4.08)		10.72 (4.35)	
Living alone	10 (7.94)	32.40 (8.53)		36.00 (3.89)		9.90 (4.98)		13.00 (6.11)	

Abbreviations: HADS, Hospital Anxiety and Depression Scale; HRQOL, health‐related quality of life; MFI‐20, Multidimensional Fatigue Index‐20; SD, standard deviation; SOMS‐7, Screening for Somatoform Symptoms‐7.

** *p*‐value < 0.01.

### 3.2 Health literacy and HRQOL

The participants' HLCCBKS scores ranged from 18 to 47 (mean = 34.19, SD = 6.18). The most common mistakes were made in Part 1 (regarding which diseases can be caused by passive smoking) and Part 3 (regarding the meaning of “OTC” on pill box), with correct rates being 23.4% and 26%, respectively.

The mean total score of participants' MFI‐20 was 37.18 (SD = 4.59; range, 27–48), indicating a medium level of fatigue. The mean score of the HADS was 10.28 (SD = 4.14; range 2–21), which confirms the existence of anxiety and depression in DTC patients. And the mean score of SOMS‐7 was 12.41 (SD = 5.15; range 1–26). The common symptoms in female were arthralgia (78.57%), weakness and fatigue (57.14%) and menoxenia (28.57%); whereas in male, the common symptoms were arthralgia (35.71%), weakness and fatigue (29.37%) and erectile dysfunction or premature ejaculation (19.84%).

### 3.3 Relationships among health literacy associated with demographics, disease variables, health literacy, and HRQOL factors

The health literacy was found significantly associated with the employment status of the participants. The participants who were currently working at the time of interview showed a higher level of health literacy compared with those unemployed (*t* = 3.17, *p* = 0.002). While in regarding to age, gender, marital status, education level, living arrangement, and frequency of having DTC education, no significant association with health literacy was identified (Table 1).

#### 3.3.1 Factors associated with fatigue level

The DTC patients' score of MFI‐20 was not correlated with the sociodemographic characteristics as described in Table 1. The Pearson correlation analysis showed that fatigue level was negatively associated with DTC education (*r* = −0.290, *p* < 0.001) as well as health literacy (*r* = −0.329, *p* < 0.001, Table 2). The stepwise regression model with MFI‐20 score as a dependent variable and DTC education, and health literacy as predictors were performed which showed that health literacy and receiving DTC education were significant variables associated with fatigue level, accounting for 16.2% of the variance (*F* = 13.13, *p* < 0.001, Table 3). Patients, who had higher level of health literacy and received more DTC‐related education, were less fatigue than those who showed lower level of health literacy and received less DTC‐related education. Health literacy explained 10.1% variance in fatigue level, and DTC‐related education explained the rest part (6.1%). The demographic characteristics, including age, gender, marital status, education level, employment status and living arrangement, were excluded from the regression mode.

**Table 2:** Person product–moment correlation coefficients among study variables

	Age	DTC education	Health literacy	MFI‐20 score	HADS score	SOMS‐7 score
Age	1					
DTC education	0.026	1				
Health literacy	0.020	0.094	1			
MFI‐20 score	0.001	−0.290**	−0.329***	1		
HADS score	−0.054	−0.253**	−0.508***	0.367***	1	
SOMS‐7 score	0.300**	−0.047	−0.052	0.134	−0.019	1

Abbreviations: DTC, Differentiated thyroid carcinoma; DTC education: number of DTC‐related teaching classes received in the past 12 months; HADS, Hospital Anxiety and Depression Scale; MFI‐20, Multidimensional Fatigue Index‐20; SOMS‐7, Screening for Somatoform Symptoms‐7.

* *p*‐value < 0.05

** *p*‐value < 0.01

*** *p*‐value < 0.001.

**Table 3:** Results of stepwise regressions on HRQOL among DTC patients

Model	Dependent variables	Independent variables	*β*	*R* ^2^	Adjusted *R* ^2^	*F*	VIF
1	MFI‐20 score	Health literacy	−0.305***	0.176	0.162	13.13***	1.01
		DTC education	−0.261**				1.01
2	HADS score	Health literacy	−0.435***	0.327	0.310	19.74***	1.12
		MIF‐20 score	0.177*				1.21
		DTC education	−0.161*				1.09
3	SOMS‐7 score	Age	0.300**	0.090	0.083	12.27**	1.00

Abbreviations: DTC, Differentiated thyroid carcinoma; DTC education, number of DTC‐related teaching classes received in the past 12 months; HADS, Hospital Anxiety and Depression Scale; HRQOL, health‐related quality of life; MFI‐20, Multidimensional Fatigue Index‐20; SOMS‐7, Screening for Somatoform Symptoms‐7.

* *p*‐value < 0.05

** *p*‐value < 0.01

*** *p*‐value < 0.001.

#### 3.3.2 Factors associated with anxiety and depression

No association was detected between the demographic characteristics (gender, marital status, education level, employment status, and living arrangement) with anxiety and depression (HADS scores) of DTC patients (Table 1). Pearson correlation analysis showed that anxiety and depression were negatively correlated with DTC‐related education (*r* = −0.253, *p* = 0.004) and health literacy (*r* = −0.508, *p* < 0.001). On the other hand, it was positively correlated with fatigue level (*r* = 0.367, *p* < 0.001), indicating the higher level of anxiety and depression correlated with a higher level of fatigue level (Table 2). The stepwise regression analysis identified health literacy, fatigue level and DTC‐related education as significant variables associated with anxiety and depression of DTC patients, which explained 31.0% of the variance (*F* = 19.74, *p* < 0.001, Table 3). Patients who had higher health literacy, received more DTC‐related education and less fatigue were less anxious and depressive than those who had less health literacy, DTC‐related education and who were more fatigable. Of note, most of the variance (25.2%) in anxiety and depression was explained by health literacy, followed by fatigue level and DTC‐related education accounted for 4.0% and 1.8% of the variance, respectively.

#### 3.3.3 Factors associated with somatoform symptoms

The basic demographics characteristics were not associated with DTC patients' somatoform symptoms, which was shown in Table 1. The age of patients (*r* = 0.300, *p* = 0.001) were positively correlated with the SOMS‐7 scores (Table 2). And the stepwise regression model with SOMS‐7 scores as the dependent variable was performed, revealing age was significant variable associated with somatoform symptoms, accounting for 8.3% of the variance in DTC patients (*F* = 12.27, *p* = 0.001, Table 3). It indicates that older patients complained of more somatoform symptoms than younger patients. Other demographic characteristics (gender, marital status, education level, employment status, living arrangement), fatigue level and anxiety/depression status were excluded from the regression mode.

## 4 DISCUSSION

The current research evaluated the association between health literacy and HRQOL in patients with differentiated thyroid carcinoma using multiple HRQOL parameters. There was a higher risk of fatigue and anxiety/depression impairments in DTC patients with a lower health literacy level across the entire sample. Thus, after adjusting for sociodemographic characteristics, a negative correlation between health literacy and HRQOL was identified in DTC patients.

The mean score (34.19, SD = 6.18) of health literacy represented a limited health literacy of the sample (Chinese Ministry of Health 2009), which supported a previous finding that the basic health literacy among Chinese residents was limited from low to moderate.^22^, ^34^, ^35^ Previous research revealed that older patients were associated with lower health literacy,^36^ and age was identified as a factor that need to be considered when reading comprehension was required for certain health or medical tasks. However, this trend was not observed in our study. The possible reason could be attributed to that a number of older patients were not included in the sample with a mean age of our participants was 43.32 years old (SD = 11.31; range = 16–65). Since the older age was the greatest risk factor for Alzheimer's disease^37^ and was associated with cognitive impairment,^38^ we excluded older patients with various degrees of psychiatric illness or dementias which may impair their cognition. Hence, the age factor didn't influence the result of health literacy.

A recent population‐based survey in south China reported different occupations were significantly associated with health literacy.^35^ In addition to that finding, we found that unemployed participants had lower health literacy than those who were employed. It's probably because the employed participants are more likely to access medical outreach programs and thyroid diseases screening tests in mainland China through health examination organized by employers. No statistically significant differences were identified in levels of health literacy among participants of different educational levels, which was not consistent with the previous study.^35^ This may reflect the lack of variation in the education levels of the current sample.

We also observed that the fatigue level and anxiety/depression status of DTC patients were both negatively associated with health literacy and DTC‐related education. Meanwhile, the anxiety/depression status was positively correlated with fatigue level. This suggests that DTC‐related education and health literacy maybe important predictors for DTC patients' HRQOL, and the physical or mental fatigue caused by surgery, radioiodide ablation or DTC itself may increase the anxious and/or depressive feelings. When making medical decisions, clinicians should consider the patients' literacy levels and received DTC‐related education to enhance the compliance of DTC patients. Since good patient education is one of the key factors to positive outcomes,^39^ it's important to fully communicate with DTC patients, improve patients' accessibility to DTC‐related education in China, and obtain better understanding and cooperation throughout the prevention, treatment and recovery. However, there were still a marked proportion of our samples (5.56%) revealing that a lack of knowledge of DTC or risk factors which are normally obtained from healthcare professionals. This reflects that extra efforts are needed to redesign the DTC‐related information to be more accessible, understandable, and relevant to individuals with different levels of health literacy, and provide basic patient education in a more systemic way.

Our results also showed that DTC patients' somatoform symptoms were only positively associated with age. Older patients had more somatoform symptoms than younger patients, and we found no direct relationship between somatoform symptoms with DTC‐related education, health literacy and other HRQOL index. This result suggests that all the somatoform symptoms of DTC patients may not only be caused by thyroid tumor or related medical interventions, but may also be caused by age‐associated chronic diseases, such as hypertension, Alzheimer's disease, diabetes, rheumatoid arthritis, etc. In addition, since our study included participants with a broad age range and generally middle‐aged patients, further studies which proportionally recruit representing different levels of age may offer some more insights into the relationships between health literacy and HRQOL among individuals with DTC.

## 5 CONCLUSIONS AND RELEVANCE TO CLINICAL PRACTICE

In summary, our study highlights a positive correlation between the level of health literacy, DTC‐related education among individuals with DTC and the HRQOL in DTC patients regarding to fatigue level and anxiety/depression status. It also reveals the patients' health literacy plays an important role in improving HRQOL. DTC education materials are required to be more understandable to low education level patients and more accessible to unemployed populations. To improve the HRQOL of DTC patients, a broader community should be included in the outreach health activities and thyroid diseases screening programs.

## AUTHOR CONTRIBUTIONS

**MengMeng Huang**: Conceptualization; data curation; formal analysis; investigation; methodology; visualization; writing — original draft; writing — review & editing. **YunJian Wu**: Conceptualization; data curation; formal analysis; methodology; writing — review & editing. **XianXiu Wen**: Conceptualization; project administration; resources; supervision; writing — review & editing. **WenZhong Song**: Methodology; project administration; resources.

## CONFLICTS OF INTEREST

Some figures in this paper were created with the help of BioRender software. The authors declare no conflicts of interest.

## TRANSPARENCY STATEMENT

The lead author XianXiu Wen affirms that this manuscript is an honest, accurate, and transparent account of the study being reported; that no important aspects of the study have been omitted; and that any discrepancies from the study as planned (and, if relevant, registered) have been explained.

## Data Availability

The data that support the findings of this study are available from the corresponding author upon reasonable request. Restrictions apply to the availability of these data, which were used under license for this study.
